# Molar Incisor Hypomineralization and Related Risk Factors among Primary School Children in Jeddah: A Cross-Sectional Study

**DOI:** 10.3390/children11101224

**Published:** 2024-10-09

**Authors:** Khlood Baghlaf, Ghazal Abdulhadi Bokhari, Fatmah Yousef Aljehani, Raneem T. Shaker, Maha Alshehri, Abdullah Almushali, Abdulaziz Alharthi, Heba Jafar Sabbagh

**Affiliations:** 1 Pediatric Dentistry Department, Faculty of Dentistry, King Abdulaziz University, Jeddah 21589, Saudi Arabia; kbaghlaf@kau.edu.sa (K.B.); mraalshehri1@kau.edu.sa (M.A.); 2 Faculty of Dentistry, King Abdulaziz University, Jeddah 21589, Saudi Arabia; gbukhari0009@stu.kau.edu.sa (G.A.B.); faljehani0039@stu.kau.edu.sa (F.Y.A.); rshaker0006@stu.kau.edu.sa (R.T.S.); aalmushali@stu.kau.edu.sa (A.A.); aalharthi0457@stu.kau.edu.sa (A.A.)

**Keywords:** etiology, hypomineralization, MIH, postnatal, pre-natal, risk factors

## Abstract

Background/Objectives: Molar Incisor Hypomineralization (MIH) is a prevalent multifactorial developmental dental defect with unclear etiology. This study aimed to identify potential risk factors for MIH among primary school children in Jeddah, Saudi Arabia. Methods: A cross-sectional study including children aged 7 to 10 years old, recruited from twelve randomly selected schools located in three regions of Jeddah city, was carried out. The children were examined for MIH and dental caries using the European Academy of Pediatric Dentistry Criteria (2003) and the DMFT/dmft WHO index. Parents were asked to provide medical and socio-demographic information on their children from their first two years of life. Results: A total of 2010 children were examined, with 888 parents responding to the survey (a 44.2% response rate). Lower maternal education was found to be significantly associated with a decreased adjusted odds ratio (AOR) of MIH (Model 1: *p* = 0.014, AOR = 0.646, and 95% CI = 0.456 to 0.915; Model 2: *p* = 0.019, AOR = 0.658, and 95% CI = 0.465 to 0.933). Being an only child, a child’s birth order, type of delivery, breastfeeding, and medical conditions were not associated with MIH. Conclusions: The significant association between MIH and maternal education suggests that further research is needed to explore the underlying mechanisms and identify other potential confounding variables.

## 1. Introduction

Enamel is the hard outer coating layer of the teeth, which undergoes a prolonged duration of development in a human’s lifespan. During this duration, various systemic and local environmental elements can influence the developmental process for teeth, leading to changes or damage in the formation of the enamel organ [1]. Furthermore, the process of odontogenesis, which continues to influence the teeth from birth until adulthood, begins in the uterus and continues until around 18–25 years later [2]. Because of this, enamel irregularities and defects can be caused by a wide variety of irritations [3].

One of the most common enamel irregularities and defects that affects the permanent incisors and one or more of the permanent first molars is Molar Incisor Hypomineralization (MIH) [4]. Worldwide, MIH has been recognized by dental clinicians as an increasingly significant issue. Various articles from multiple countries have looked at the epidemiology of MIH [5]. A systematic review including twenty-four studies worldwide showed a prevalence of MIH ranging between 2.4 and 40.2% [5]. In the Middle East, a recent systematic review included 29 studies and found the mean prevalence of MIH was 15%, which is comparable to its prevalence globally [6].

Multiple scoring systems are available for diagnosing MIH, such as the European Academy of Pediatric Dentistry criteria [7], the developmental defects of enamel index (the DDE index), its modified version (the mDDE index) [8], and the Enamel Defects Index (EDI) [9]. The European Academy of Pediatric Dentistry (EAPD) established its diagnostic criteria for MIH in 2003. These criteria include demarcated opacities, post-eruptive enamel breakdown (PEB), atypical restoration, atypical caries, extracted molars due to MIH, and failure of eruption of a molar or an incisor [7].

The etiology of MIH is multifactorial, resulting from a combination of genetic susceptibility, environmental influences, and disturbances in tooth formation [10]. Based on a recent systematic review conducted by Garot et al. that included 45 studies, unspecified maternal illnesses, prematurity, and caesarean delivery were associated with an increased risk of developing MIH [10]. This systematic review found postnatal factors including measles, urinary tract infections, otitis media, gastric disorders, bronchitis, kidney diseases, pneumonia, and asthma to be associated with MIH. Other factors, such as fever, antibiotic use, and genetic factors, were also associated with MIH [10].

Several studies have found associations between specific illnesses such as asthma, fever, or adenoid infections and MIH in early childhood [11,12]. These conditions might negatively impact the process of enamel mineralization through the direct effects of the disease or due to factors like a lack of oxygen, low calcium levels, and malnutrition [13].

Few studies in Saudi Arabia have studied the risk factors for MIH among primary school children [14,15]. Therefore, this cross-sectional study aimed to evaluate the potential risk factors for MIH, including socioeconomic variables, pregnancy difficulties, and medical conditions experienced by children during their first two years of life.

## 2. Materials and Methods

### 2.1. Ethical Considerations

This study adhered to the Declaration of Helsinki guidelines; ethical approval was obtained from the Research Ethics Committee, the Faculty of Dentistry, King Abdulaziz University (No. 175-11-23); and consent forms were signed by parents to approve their children’s participation in the study.

### 2.2. Study Design

This was a cross-sectional study targeting 7–10-year-old school children in second, third, fourth, and fifth grade attending governmental primary schools in Jeddah, Saudi Arabia, from the period of October 2023 to June 2024.

We were provided with a list of schools by the Ministry of Education from each area in Jeddah (south, north, and central) and an approval letter regarding facilitating the researchers’ mission to visit the schools. Consent forms were signed by all parents before they participated in the study.

The study design involved two parts: the first part involved clinical examination during primary school visits (using the DMFT and dmft index and the MIH criteria by the European Academy of Pediatric Dentistry); the second part was a validated questionnaire [16] that was submitted to the children’s parents, which included their demographic characteristics, medical history, feeding history, and exposure to medications during their first 2 years of life.

### 2.3. Sample Size and Sampling Technique

The sample size calculation for the study revealed that 1574 responses were needed according to a sample power of 85%, using openEpi for each school section. We referred to Bukhari et al. 2023 in hypothesizing that there was a relationship between the risk factors they presented and the presence of MIH [6].

### 2.4. Sample Selection for the Study

The study included 7–10-year-old children in second, third, fourth, and fifth grade attending governmental primary schools in Jeddah, Saudi Arabia. The schools were selected using stratified random sampling. We divided the population based on the area of the schools (southern, northern, and central), and for each area, we randomly selected 2 schools using computer number generator methods. In cases where some schools were not accessible, we selected more schools randomly from the same area.

### 2.5. Inclusion and Exclusion Criteria

Every child who met the following criteria was included:

Inclusion criteria:

- Male and female children aged 7–10 years old.
- Children of all nationalities (Saudis and non-Saudis).
- Children who lived in Jeddah city.
- Children in whom at least one first permanent molar had erupted.

Exclusion criteria: Children with orthodontic brackets, those with no molars or whose molars are covered with crowns, and uncooperative children.

### 2.6. Data Collection Methods

#### 2.6.1. Phase 1: Clinical Examination during School Visits

Before starting the dental examinations in the schools, the examiners were trained to diagnose MIH using photographs of different presentations of the condition. A group of 10 patients aged 7–10 years old who were not part of the study sample was used for calibration before the commencement of the data collection for the study (Cohen’s kappa coefficient = 0.875).

The process began with meticulous planning, in which suitable times and areas within the school were arranged in collaboration with the school principals and health counselors. All printed documents necessary for the examination were organized, and disposable examination kits equipped with gloves were prepared in advance to ensure a smooth process. Before the examination, the students were gently briefed on what to expect in order to alleviate any potential anxiety they might have experienced.

Each dental examination was conducted individually, adhering to strict hygiene protocols and utilizing disposable examination kits comprising dental mirrors, probes, and gloves. The students were asked to sit comfortably on a designated chair, ensuring proper positioning for the examination. A comprehensive assessment was carried out, examining all of their primary and permanent teeth and surfaces in a standardized order. Caries status was recorded using the criteria outlined by the World Health Organization (WHO), employing the indices “Decayed, Missing, and Filled Teeth/Surfaces” (DMFT/DMFTS) for permanent teeth and “decayed, missing, and filled teeth/surfaces” (dmft/dmfts) for primary teeth [17]. Additionally, attention was paid to identifying any enamel defects during the examination process.

Following the completion of each student’s examination, a designed questionnaire was provided to them, intended for completion by their parents or guardians. This questionnaire aimed to gather further insights into the students’ oral health practices and any relevant medical history, ensuring a comprehensive understanding of their dental needs. Concluding the visit, a concise yet informative educational lecture was delivered to the students, emphasizing the importance of oral health maintenance practices, such as regular brushing with fluoride toothpaste, flossing, and reducing their consumption of sweets and sugary drinks. This educational segment served as a proactive measure to empower students with the knowledge and skills necessary to maintain optimal dental health and prevent dental caries.

#### 2.6.2. Phase 2: A Validated Questionnaire

As part of this study, we constructed a hard/soft copy of a questionnaire for parents/caregivers regarding the participants’ underlying medical conditions. This questionnaire was carefully built to identify all possible etiological conditions associated with MIH and related to a child’s medical history during their first two years of life, as well as the mother’s pregnancy history. The questionnaire included general information about the study group, the name of the city, and the school’s name. The questions about their demographic characteristics included the child’s age, gender, place of birth, and residence, as well as their parents’ education, age at the time of the child’s delivery, and income; the type of delivery; and their feeding history, medical history and exposure to medications, illnesses, and infections during their first two years of life. The questionnaire included questions about medical conditions that were associated with MIH in the systematic review by Bukhari. et al., 2022 [6]. Then, the questionnaire was translated into the Arabic language. The validity of the questionnaire was evaluated by five experts in the field and by calculating the content validity index (CVI). The results showed excellent content validity, at a CVI = 0.85.

### 2.7. Statistical Analysis

The collected data were coded and entered into an Excel spreadsheet and subsequently double-checked to verify their accuracy. The analysis of the data was carried out using Statistical Package for Social Sciences computer software (SPSS 18.0, Inc., Chicago, IL, USA). Means, standard deviation, and t-tests were used to assess continuous data such as parental age. Frequencies, percentages, and Chi-square tests were used to evaluate nominal data. A probability value of less than 0.05 was regarded as statistically significant. Two regression models were employed to analyze the dependent variable (the child affected by MIH) and its related risk factors. Model 1 included the mother’s age, the child’s sex, the mother’s education level, whether the child was an only child or had siblings, the type of delivery, breastfeeding, and any medical conditions. Model 2 included the same independent variables except that the “only child” variable was replaced with the child’s birth order.

## 3. Results

### 3.1. Demographic Characteristics of the Sample

This study was divided into two phases; in phase 1, a total of 2010 children from twelve randomly selected schools consented and participated in this study, comprising 945 males (47%) and 1065 females (53%). MIH was observed in 359 children (prevalence: 17.9%). Phase 2 involved the distribution of the questionnaire and parents responding to this questionnaire. A total of 888 parents responded, resulting in a response rate of 44.2%. The mean age of the children included was 8.78 years (±0.975). Among the respondents, the prevalence of MIH was 160 out of 888 (18.0%).

Table 1 presents the demographic characteristics of the participants. The sample included 333 males (37.5%) and 555 females (62.5%), with a statistically significant difference between the two groups (*p* = 0.003). The prevalence of MIH was significantly higher among children whose mothers had a higher educational level (90 children, 56.2%) compared to those whose mothers had a lower educational level (70 children, 43.8%) (*p* = 0.013). Furthermore, MIH was more common among first- or second-born children (99 children, 62.7%) compared to those with a higher birth order (59 children, 37.3%), although this difference was not statistically significant (*p* = 0.065).

### 3.2. Birth Difficulties, Breastfeeding Practices, and Exposure to Medical Conditions during the First Two Years of Life

Table 2 shows the distribution of the participants based on birth difficulties, breastfeeding practices, and exposure to medical conditions during their first two years of life. In the MIH group, 2 children (1.2%) had reportedly experienced birth difficulties, 90 children (56.2%) had medical conditions, and 13 children (8.1%) were hospitalized during their first two years of life. In the non-MIH group, the corresponding figures were 23 children (3.2%), 435 children (59.8%), and 92 children (12.6%), respectively. However, these associations were not statistically significant (*p* = 0.186, *p* = 1.05, and *p* = 0.109, respectively). Similarly, Table 3 indicates that the prevalence of MIH was high among children with a limited duration of breastfeeding (less than 12 months). However, the relationship between the duration of breastfeeding and the occurrence of MIH was not significant (*p* = 0.981).

### 3.3. The Regression Analysis for Risk Factors Associated with MIH

Table 4 shows the results of the regression analysis for risk factors associated with MIH. Paternal education and family socioeconomic status were excluded from the analysis to avoid collinearity. A lower level of maternal education was found to be significantly associated with a decreased adjusted odds ratio (AOR) of MIH (Model 1: *p* = 0.011, AOR = 0.638, and 95% CI = 0.450 to 0.903; Model 2: *p* = 0.019, AOR = 0.658, and 95% CI = 0.465 to 0.933). Additionally, being an only child showed a non-statistically significant tendency to increase the AOR of having MIH (*p* = 0.144, AOR = 1.713, 95% CI: 0.832 to 3.526). Additionally, child’s birth order, type of delivery, breastfeeding, and medical conditions were not associated with MIH.

## 4. Discussion

This cross-sectional study was conducted in Jeddah city to examine the association between MIH and potential risk factors for MIH, including socioeconomic variables, pregnancy difficulties, and medical conditions. A validated questionnaire was sent to each child’s parent. The age range of 7 to 10 years old was chosen mainly because every child has at least one erupted first molar in this age range. A wide range of clinical manifestations may arise from anomalies in the enamel and dentin since many factors can influence how hard tooth tissues form [3]. Lesions can be localized, impacting just one or two teeth, or they can be generalized, affecting several teeth or the complete dentition. Furthermore, these flaws can be distributed symmetrically or asymmetrically throughout the dental arch [3].

Our findings showed that the prevalence of MIH among primary school children in Jeddah city was 17.9%, which was higher but comparable to the mean prevalence of MIH in the Middle East (15%) based on a recent systematic review [6] in Riyadh, Saudi Arabia, reporting a prevalence of MIH of 15.2%. A study conducted in Iraq reported a similar MIH prevalence of 18.6% [12], while the previous Saudi review found no significant difference in the prevalence between the genders [15].

The results of this study were consistent with the findings of the former systematic review regarding the gender distribution of MIH. Our study found that MIH was more prevalent among girls than boys. A recent systematic review by [18] included 59 cross-sectional studies and showed the total number of girls described by most studies was 8861 (mean: 466), of which 1062 (mean: 59) had MIH, while the total number of boys was 8203 (mean: 432), of which 972 (mean: 54) had MIH [18]. However, the gender distribution was not mentioned by any of the studies included in this systematic review.

The regression analysis showed an association between MIH and highly educated mothers. Highly educated mothers are usually liable to stress due to their working lifestyle, and several studies have found an association between maternal stress and MIH [19]. Based on a systematic review that included 29 studies, maternal psychological stress (OR = 2.65; 95% CI = 1.52–4.63; *p* = 0.001) was observed to be significantly associated with a higher odds of MIH. However, a previous study conducted in Mexico City showed that the prevalence of MIH was also high among mothers with elementary school education or some middle school education [20].

The study presented revealed a high prevalence of MIH among 102 children (63.8%) who had also had a postnatal fever. However, this difference was not statistically significant (*p* > 0.05). Furthermore, based on the results of the systematic review [6], six studies out of seven found high fever to be associated with the presence of MIH. Another systematic review including 64 studies [10] found an association between MIH and fever, which may be considered a consequence of childhood illnesses.

Previous studies have revealed a significant correlation between early childhood illnesses, such as ear infections, respiratory distress, and a history of tonsillitis, and the occurrence of MIH [10,21]. These illnesses were determined to be major indicators of MIH. However, in our findings, no significant association was found between pregnancy-related issues or childhood illnesses and the presence of MIH, which is in agreement with several cross-sectional studies [13,22].

Children affected with otitis media or tonsillitis usually experience a high fever and are frequently given antibiotics as treatment. The present study found no significant link between high fever and antibiotic intake and the presence of MIH. However, prior studies [10,13] have reported significant associations. One study [23] found that the administration of amoxicillin in the early stages of life could disrupt the process of mineralization, leading to the development of MIH [23]. Another study mentioned that amoxicillin might alter the expression of some genes essential to enamel development and reported that high fever altered the stage of enamel matrix formation and enamel mineralization [23].

In our study, MIH was prevalent (51%) among the group with a limited duration of breastfeeding (less than 12 months), but no significant association was found between breastfeeding duration and MIH. Similar findings have shown that breastfeeding might influence the development of MIH if the period of breastfeeding does not exceed 12 months [10,24,25].

Recent research indicates that MIH may arise from an interplay of systemic and genetic variables [10]. One study revealed that 12.2% of individuals affected by MIH had no pertinent medical background [26]. This finding provides weight to the concept that genetic factors may contribute to the development of the condition [10].

This study demonstrated several strengths, such as a large sample size of primary school children that were living in the same environment. Additionally, the use of stratified random sampling, dividing the population based on the area of schools (southern, northern, and central), is considered a strength of this study. In terms of generalizability of the results and overcoming publication bias, the distribution of MIH among the participants who responded to the questionnaire (18%) was similar to its frequency among the total sample (17.9%).

One limitation that might have affected our study is recall bias. Even with extensive questioning, the collected data may not fully represent the children’s medical history over their first two years of life. Further studies can overcome this issue using children’s medical records. This may enhance the quality of the medical information obtained from children’s parents. However, this approach may not include information on minor illnesses or management not advised by a physician, such as the use of over-the-counter medications.

In addition, there are a few other limitations to the current study that should be discussed as well. The most suitable period for evaluating defects in the development of the enamel is shortly after the teeth have erupted, as these findings are not stable and can be lost due to dental caries, trauma, or attrition. Moreover, accessing the schools was challenging due to unresponsive administrations, frequent holidays, and student absences before and after holidays.

## 5. Conclusions

The prevalence of MIH among this population of children was 18%. The response rate was relatively low. This study suggests that maternal education, living with both parents, and having siblings may play a role in the etiology of MIH. Additional studies are needed to explore the underlying reasons for this association and to investigate other related potential contributing factors.

## Figures and Tables

**Table 1:** Distribution of the sample according to participants’ socio-demographic data. N = 888.

Variables		Is There MIH?		Total N (%)	*p* Value
		Yes N (%)	No N (%)		
Child’s gender	Male	55 (34.4)	278 (38.2)	333 (37.5)	0.003 *^c^
	Female	105 (65.6)	450 (61.8)	555 (62.5)	
Child’s age	Mean ± SD	8.69 ± 0.95	8.76 ± 0.97	8.75 ± 0.965	0.420 ^t^
Mother’s age ^R^	≤25	81 (50.6)	338 (46.4)	419 (47.2)	0.336 ^c^
	>25	79 (49.4)	390 (53.6)	469 (52.8)	
Father’s age ^R^	≤25	53 (33.1)	190 (26.1)	243 (27.4)	0.071 ^c^
	>25	107 (66.9)	538 (73.9)	645 (72.6)	
Parenting type	Single parent	8 (5.0)	45 (6.2)	53 (6.0)	0.565 ^c^
	Both parents	152 (95.0)	682 (93.8)	834 (94.0)	
Mother’s education	≤High school	70 (43.8)	397 (54.5)	467 (52.6)	0.013 *^c^
	>High school	90 (56.2)	331 (45.5)	421 (47.4)	
Father’s education	≤High school	76 (47.5)	369 (50.7)	445 (50.1)	0.465 ^c^
	>High school	84 (52.5)	359 (49.3)	443 (49.9)	
Family income	Low	53 (33.1)	265 (36.4)	318 (35.8)	0.202 ^c^
	Moderate	95 (59.4)	382 (52.5)	477 (53.7)	
	High	12 (7.5)	81 (11.1)	93 (10.5)	
Is the child an only child?	Yes	11 (6.9)	29 (4.0)	40 (4.5)	0.624 ^c^
	No	149 (93.1)	699 (96.0)	848 (95.4)	
What is the child’s birth order?	First or second child	99 (62.7)	396 (54.6)	495 (56.1)	0.065 ^c^
	Higher than second-born	59 (37.3)	329 (45.4)	388 (43.9)	

MIH: Molar Incisor Hypomineralization. C: Chi-square test. SD: Standard deviation.Family income: Low: “in debt” or “just meets routine expenses”; Moderate: “meets routine and emergency expenses”; high: “able to save and invest money”. ^R^ Parental age at time of child’s delivery.^t^ *t*-test; ^c^ Chi-square test; * significant at *p* = 0.05.

**Table 2:** Distribution of the sample according to children’s birth difficulties, breastfeeding, and their exposure to medical conditions during their first two years of life.

Variables		Is There MIH_MIH Only?			*p* Value
		Yes N (%)	No N (%)	Total N (%)	
Caesarean vs. normal birth	Caesarean	54 (33.8)	237 (32.6)	291 (32.8)	0.771 ^c^
	Normal delivery	106 (66.2)	491 (67.4)	597 (67.2)	
Was the pregnancy difficult?	Yes	2 (1.2)	23 (3.2)	25 (2.8)	0.186 ^b^
	No	158 (98.8)	705 (96.8)	863 (97.2)	
Was the baby breastfed?	Yes	142 (88.8)	654 (89.8)	796 (79.5)	0.964 ^c^
	No	18 (11.2)	74 (10.2)	92 (20.5)	
Did the child have any medical condition during their first 2 years of life?	Yes	90 (56.2)	435 (59.8)	525 (59.1)	0.105 ^c^
	No	70 (43.8)	293 (40.2)	363 (40.9)	
Was the child hospitalized during their first 2 years of life?	Yes	13 (8.1)	92 (12.6)	105 (11.8)	0.109 ^c^
	No	147 (91.9)	636 (87.4)	783 (88.2)	
Types of medical conditions					
Fever	Yes	102 (63.8)	498 (68.4)	600 (67.6)	0.255 ^c^
	No	58 (36.2)	230 (31.6)	288 (32.4)	
Use of antibiotics	Yes	58 (36.2)	247 (33.9)	305 (34.3)	0.851 ^c^
	No	102 (63.8)	481 (66.1)	565 (63.6)	
Asthma and allergies	Yes	23 (14.4)	133 (18.3)	156 (17.6)	0.24 ^c^
	No	137 (85.6)	595 (81.7)	732 (82.4)	
Pneumonia	Yes	0 (0.0)	40 (5.5)	40 (4.5)	0.002 ^b^*
	No	160 (100.0)	688 (94.5)	848 (95.5)	
Jaundice	Yes	0 (0.0)	6 (0.8)	6 (0.7)	0.249 ^b^
	No	160 (100.0)	722 (99.2)	882 (99.3)	
Congenital hereditary disease	Yes	0 (0.0)	16 (2.2)	16 (1.8)	0.058 ^b^
	No	160 (100.0)	712 (97.8)	872 (98.2)	
Any ENT problem	Yes	13 (8.1)	55 (7.6)	68 (7.7)	0.806 ^c^
	No	147 (91.9)	673 (92.4)	820 (92.3)	
Skin abnormalities	Yes	1 (0.6)	9 (1.2)	10 (1.1)	0.507 ^b^
	No	159 (99.4)	709 (97.4)	878 (98.9)	
Speech/hearing problems	Yes	1 (0.6)	19 (2.6)	20 (2.3)	0.125 ^b^
	No	159 (99.4)	719 (98.8)	868 (97.7)	
Mental problems	Yes	1 (0.6)	0 (0.0)	1 (0.1)	0.033 *^b^
	No	157 (99.4)	721 (100.0)	878 (99.9)	

MIH: Molar Incisor Hypomineralization. Family income: Low: “in debt” or “just meets routine expenses”; Moderate: “meets routine and emergency expenses”; high: “able to save and invest money”. ^c^ Chi-square test; ^b^ Fisher’s exact test; * significant at *p* = 0.05.

**Table 3:** Distribution of the sample according to Molar Incisor Hypomineralization and the duration of breast feeding.

Variables		Is There MIH?		Total N (%)
		Yes N (%)	No N (%)	
Duration of breastfeeding	No	18 (11.2)	74 (10.2)	92 (10.4)
	1–3 months	25 (15.6)	106 (14.6)	131 (14.8)
	6 months	36 (22.5)	169 (23.2)	205 (23.1)
	12 months	23 (14.4)	114 (15.7)	137 (15.4)
	>12 months	58 (36.2)	265 (36.4)	323 (36.4)
Total		160 (100.0)	728 (100.0)	888 (100.0)

*p* value = 0.981. MIH: Molar Incisor Hypomineralization

**Table 4:** Regression analysis for risk factors affecting Molar Incisor Hypomineralization.

Variables		Model 1	Model 2
		*p* Value, AOR (95% CI)	*p* Value, AOR (95% CI)
Gender	Male	0.577, 0.894 (0.603–1.326)	0.552, 0.887 (0.598–1.316)
	Female	1	1
Mother’s age	≤25	0.382, 1.168 (0.825–1.653)	0.553, 1.114 (0.780–1.590)
	>25	1	1
Mother’s education	≤High school	0.011, 0.638 (0.450–0.903)	0.019, 0.658 (0.465–0.933)
	>High school	1	1
Are they an only child?	Yes	0.144, 1.713 (0.832–3.526)	
	No	1	
What is the child’s birth order?	First- or second-born		0.103, 1.355 (0.940–1.954)
	Higher than second-born		1
Type of delivery	Caesarean	0.813, 1.046 (0.722–1.515)	0.725, 1.069 (0.738–1.549)
	Normal delivery	1	1
Was the baby breastfed?	Yes	0.909, 0.968 (0.554–1.691)	0.970, 0.989 (0.566–1.730)
	No	1	1
Medical conditions?	Yes	0.522, 0.884 (0.605–1.291)	0.547, 0.890 (0.609–1.300)
	No	1	1

AOR: Adjusted Odds Ratio. Low: “in debt” or “just meets routine expenses”; Moderate: “meets routine and emergency expenses”; high: “able to save and invest money”.

## Data Availability

The original contributions presented in this study are included in the Supplementary Material; further inquiries can be directed to the corresponding authors.

## Supplementary Materials

The following supporting information can be downloaded at: https://www.mdpi.com/article/10.3390/children11101224/s1.

## References

[1] Drummond B.K., Kilpatrick N. (2015). Planning and Care for Children and Adolescents with Dental Enamel Defects: Etiology, Research and Contemporary Management.

[2] Peterkova R., Hovorakova M., Peterka M., Lesot H. (2014). Three-dimensional analysis of the early development of the dentition. Aust. Dent. J..

[3] Atar M., Körperich E.J. (2010). Systemic disorders and their influence on the development of dental hard tissues: A literature review. J. Dent..

[4] Rao M.H., Aluru S.C., Jayam C., Bandlapalli A., Patel N. (2016). Molar Incisor Hypomineralization. J. Contemp. Dent. Pract..

[5] Jälevik B. (2010). Prevalence and Diagnosis of Molar-Incisor- Hypomineralisation (MIH): A systematic review. Eur. Arch. Paediatr. Dent..

[6] Bukhari S.T., Alhasan H.A., Qari M.T., Sabbagh H.J., Farsi N.M. (2023). Prevalence and risk factors of molar incisor hypomineralization in the Middle East: A systematic review and meta-analysis. J. Taibah Univ. Med. Sci..

[7] Weerheijm K.L., Duggal M., Mejàre I., Papagiannoulis L., Koch G., Martens L.C., Hallonsten A.L. (2003). Judgement criteria for molar incisor hypomineralisation (MIH) in epidemiologic studies: A summary of the European meeting on MIH held in Athens, 2003. Eur. J. Paediatr. Dent..

[8] Mohamed A.R., Thomson W.M., Mackay T.D. (2010). An epidemiological comparison of Dean’s index and the Developmental Defects of Enamel (DDE) index. J. Public. Health Dent..

[9] Elcock C., Lath D.L., Luty J.D., Gallagher M.G., Abdellatif A., Bäckman B., Brook A.H. (2006). The new Enamel Defects Index: Testing and expansion. Eur. J. Oral. Sci..

[10] Garot E., Rouas P., Somani C., Taylor G.D., Wong F., Lygidakis N.A. (2022). An update of the aetiological factors involved in molar incisor hypomineralisation (MIH): A systematic review and meta-analysis. Eur. Arch. Paediatr. Dent..

[11] de Lima Mde D., Andrade M.J., Dantas-Neta N.B., Andrade N.S., Teixeira R.J., de Moura M.S., de Deus Moura Lde F. (2015). Epidemiologic Study of Molar-incisor Hypomineralization in Schoolchildren in North-eastern Brazil. Pediatr. Dent..

[12] Ghanim A., Manton D., Bailey D., Mariño R., Morgan M. (2013). Risk factors in the occurrence of molar-incisor hypomineralization amongst a group of Iraqi children. Int. J. Paediatr. Dent..

[13] Allazzam S.M., Alaki S.M., El Meligy O.A. (2014). Molar incisor hypomineralization, prevalence, and etiology. Int. J. Dent..

[14] Alhowaish L., Baidas L., Aldhubaiban M., Bello L.L., Al-Hammad N. (2021). Etiology of Molar-Incisor Hypomineralization (MIH): A Cross-Sectional Study of Saudi Children. Children.

[15] Almuallem Z., Alsuhaim A., Alqudayri A., Aljarid S., Mousa Alotaibi M., Alkraida R., Faden R., Mojaleed F., Alruwaithi M., Al-Huraishi H. (2022). Prevalence and possible aetiological factors of molar incisor hypomineralisation in Saudi children: A cross-sectional study. Saudi Dent. J..

[16] Bagher S., Alamoudi R., Trad R., Shaker R., Alamoudi R., Bader R., Sabbagh H. (2023). Medical Conditions that Could Affect Oral Health Care Among Children Living in Governmental Orphanage Centers: A Case-Control Study. J. King Abdulaziz Univ. Med. Sci..

[17] World Health Organization Oral Health Surveys: Basic Methods. https://www.who.int/publications/i/item/9789241548649.

[18] Mazur M., Corridore D., Ndokaj A., Ardan R., Vozza I., Babajko S., Jedeon K. (2023). MIH and Dental Caries in Children: A Systematic Review and Meta-Analysis. Healthcare.

[19] Patil M. (2016). Stress level of working and non working women. Int. J. Indian Psychol..

[20] Villanueva-Gutiérrez T., Irigoyen-Camacho M.E., Castaño-Seiquier A., Zepeda-Zepeda M.A., Sanchez-Pérez L., Frechero N.M. (2019). Prevalence and Severity of Molar-Incisor Hypomineralization, Maternal Education, and Dental Caries: A Cross-Sectional Study of Mexican Schoolchildren with Low Socioeconomic Status. J. Int. Soc. Prev. Community Dent..

[21] Fatturi A.L., Wambier L.M., Chibinski A.C., Assunção L., Brancher J.A., Reis A., Souza J.F. (2019). A systematic review and meta-analysis of systemic exposure associated with molar incisor hypomineralization. Community Dent. Oral. Epidemiol..

[22] Silva M.J., Scurrah K.J., Craig J.M., Manton D.J., Kilpatrick N. (2016). Etiology of molar incisor hypomineralization—A systematic review. Community Dent. Oral. Epidemiol..

[23] Laisi S., Ess A., Sahlberg C., Arvio P., Lukinmaa P.L., Alaluusua S. (2009). Amoxicillin may cause molar incisor hypomineralization. J. Dent. Res..

[24] Altner S., Milutinovic I., Bekes K. (2024). Possible Etiological Factors for the Development of Molar Incisor Hypomineralization (MIH) in Austrian Children. Dent. J..

[25] Khazaei Y., Harris C.P., Heinrich J., Standl M., Kühnisch J. (2021). Association Study on Nutrition in the First Year of Life and Molar-Incisor Hypomineralization (MIH)-Results from the GINIplus and LISA Birth Cohort Studies. Int. J. Env. Res. Public. Health.

[26] Lygidakis N.A., Dimou G., Marinou D. (2008). Molar-incisor-hypomineralisation (MIH). A retrospective clinical study in Greek children. II. Possible medical aetiological factors. Eur. Arch. Paediatr. Dent..

