# Supplementary material for: Molar Incisor Hypomineralization and Related Risk Factors among Primary School Children in Jeddah: A Cross-Sectional Study
Source: Children (Basel). 2024 Oct 9;11(10):1224. doi: 10.3390/children11101224 (PMC11506596; doi:10.3390/children11101224)
Supplement: Supplementary file 1 [file children-11-01224-s001.zip › Arabic_parent_form-MIH.pdf]

استبيان المشارك

التاريخ: ____/____/____

القسم الاول: البيانات الديموغرافية

اسم الطفل:		رقم الجوال:	
عمر الطفل:		جنس الطفل:	□ أنثى □ ذكر
الدولة:		المدينة:	
مع من يعيش الطفل؟		□ مع والديه	

○ **إذا كان الطفل يعيش مع والديه**

عمر الام	□ اقل من ٢٥ سنة □ ٢٥-٣٥ سنة □ ٣٥-٤٥ سنة □ اكثر من ٤٥ سنة
عمر الاب	□ اقل من ٣٠ سنة □ ٣٠-٤٠ سنة □ ٤٠-٥٠ سنة □ اكثر من ٥٠ سنة
مستوى تعليم الام	□ اقل من الثانوي □ تعليم ثانوي □ اعلى من الثانوي
مستوى تعليم الاب	□ اقل من الثانوي □ تعليم ثانوي □ اعلى من الثانوي
دخل العائلة الشهري	□ مدين □ بالكاد يغطي الاحتياجات الأساسية □ يغطي الاحتياجات الاساسية و الطارئة □ يستطيع الادخار و الاستثمار من دخله
هل الطفل/الطفلة الوحيد للعائلة	□ نعم □ لا
معلومات فترة الحمل والولادة	□ صعوبة في فترة الحمل □ ولادة طبيعية □ ولادة قيصرية □ ولادة مبكرة
هل تم ارضاع الطفل طبيعي	□ نعم □ لا
مدة الارضاع	□ ١-٣ شهور □ ١-٦ شهور □ ١-١٢ شهور □ اكثر من ١٢ شهر

القسم الثاني: التاريخ المرضي

هل كان لدى الطفل أي حالة مرضية خلال السنتين الأولى من عمره؟	□نعم □ لا □ لا أعلم	
**هل تم تنويم الطفل في المستشفى خلال السنتين الاولى من عمره؟**	□نعم □ لا<br>إذا كانت الإجابة نعم يرجى تحديد المشكلة ____________<br>السبب: ____________ متى؟ ____________	
**كم كان عمر الطفل حينها**	□٠-١ سنة □ ١-٢ سنة	
هل سبق أن تم تشخيصك بأي من الحالات التالية (في أول سنتين من العمر)		
اسم المشكلة الصحية		**كم مرة/ كل سنتين؟**
**ارتفاع درجة الحرارة**	□نعم □لا □لا أعلم	□١ □٢ □٣ □٤ □٥ □٦ □٧
**استخدام المضادات الحيوية**	□نعم □لا □لا أعلم	□١ □٢ □٣ □٤ □٥ □٦ □٧

الربو والحساسية	□نعم □لا □لا أعلم	□١ □٢ □٣ □٤ □٥ □٦ □٧
التهاب رئوي	□نعم □لا □لا أعلم	□١ □٢ □٣ □٤ □٥ □٦ □٧
مشاكل عقلية	□نعم □لا □لا أعلم	□١ □٢ □٣ □٤ □٥ □٦ □٧
الصرع	□نعم □لا □لا أعلم	□١ □٢ □٣ □٤ □٥ □٦ □٧
مشاكل نفسية	□نعم □لا □لا أعلم	□١ □٢ □٣ □٤ □٥ □٦ □٧
التهاب الأذن الوسطى	□نعم □لا □لا أعلم	□١ □٢ □٣ □٤ □٥ □٦ □٧
مشاكل في النطق والسمع	□نعم □لا □لا أعلم	□١ □٢ □٣ □٤ □٥ □٦ □٧
مشاكل قلبية	□نعم □لا □لا أعلم	□١ □٢ □٣ □٤ □٥ □٦ □٧
مشاكل في الكلى	□نعم □لا □لا أعلم	□١ □٢ □٣ □٤ □٥ □٦ □٧
مشاكل في الكبد	□نعم □لا □لا أعلم	□١ □٢ □٣ □٤ □٥ □٦ □٧
التهاب الغدد	□نعم □لا □لا أعلم	□١ □٢ □٣ □٤ □٥ □٦ □٧
تشوهات الجلد	□نعم □لا □لا أعلم	□١ □٢ □٣ □٤ □٥ □٦ □٧
جدري الماء / (أو) الحصبة	□نعم □لا □لا أعلم	□١ □٢ □٣ □٤ □٥ □٦ □٧
مرض وراثي أو خلقي	□نعم □لا □لا أعلم	□١ □٢ □٣ □٤ □٥ □٦ □٧
التهاب المسالك البولية	□نعم □لا □لا أعلم	□١ □٢ □٣ □٤ □٥ □٦ □٧
الحصبة الألمانية	□نعم □لا □لا أعلم	□١ □٢ □٣ □٤ □٥ □٦ □٧
اليرقان	□نعم □لا □لا أعلم	□١ □٢ □٣ □٤ □٥ □٦ □٧

**يرجى ذكر أي حالات أخرى هنا** ___________________________	□نعم □ لا<br>إذا كانت الإجابة نعم يرجى تحديد المشكلة ______________
**عدد المرات خلال السنتين الاولى من عمره؟**	□١ □٢ □٣ □٤ □٥ □٦ □٧

القسم الثالث: التاريخ المرضي المتعلق بالأسنان

الشكوى الرئيسية:	□ تسوس الأسنان □ حساسية الأسنان □ ألم الأسنان □ متابعة □للفحص
شدة الم الاسنان؟	□ بسيط □ متوسط □ شديد
هل سبق للطفل أن زار عيادة الأسنان؟	□نعم □لا □لا أعلم
هل تعرض الطفل لتخدير الأسنان سابقًا؟	□نعم □لا □لا أعلم
هل سبق للطفل أن عانى من آلم أسنان؟	□نعم □لا □لا أعلم

شكركم لمشاركتكم
