# Supplementary material for: Molar Incisor Hypomineralization and Related Risk Factors among Primary School Children in Jeddah: A Cross-Sectional Study
Source: Children (Basel). 2024 Oct 9;11(10):1224. doi: 10.3390/children11101224 (PMC11506596; doi:10.3390/children11101224)
Supplement: Supplementary file 1 [file children-11-01224-s001.zip › MHI_qs_child_MIH.pdf]

Questionnaire:

Molar-Incisor Hypomineralization (MIH) and Dental caries among children

Date:______/________/_______

Section 1: Data collector information:

**Examiner Code**	
**Child Code**	

Section 2: Child information:

**Group**	School children		
**Child name:**		**Phone Number:**	
**Child age:**		**Child gender:**	□Male □Female
**Country:**		**City:**	
**Who is this child living with?**		□ Mother □ father □Parents □others________	
**Is the child only child**		□Yes □No	
**Child order**		1 2 3 4 5 6 7 8 9	

Section3: Dental History: (Child)

**Chief complaint:**	□Decayed teeth □Sensitivity in teeth □Pain in teeth □Follow up □Check up
**Pain severity:**	□ mild □ moderate □ sever
**Did the child ever visited the dental clinic before?**	□Yes □No □I don’t know
**Did the patient experience dental anesthesia before?**	□Yes □No □I don’t know
**Has the child ever had toothache in the past?**	□Yes □No □I don’t know
**If yes:**<br>**The pain is related to (you can select more than one**	□Cold<br>□Hot<br>□While eating<br>□ spontaneous

Section 4: Clinical Examinations.

Kindly, use the WHO scoring system to record DMFS/dmfs below, then use MIH/HSPM recording sheet. (Conducted by EAPD) to record MIH/HSPM accordingly.

A. DMFs score

Condition	Dmfs codes	DMFS codes
Sound	A	0
decayed	B	1
Missing due to caries	C	2
Filled	D	3
Pit and fissure sealant	E	4

Primary dentition:

55	54	53	52	51	61	62	63	64	65
85	84	83	82	81	71	72	73	74	75

ds:__ ms:__ fs:__ dmfs score-: ___

Permanent dentition:

18	17	16	15	14	13	12	11	21	22	23	24	25	26	27	28
48	47	46	45	44	43	42	41	31	32	33	34	35	36	37	38

DS:__ MS:__ FS:__ DMFS score-: ___

B. MIH Score/HSPM

Kindly, use the EAPD criteria to diagnose enamel defect respectively

Kindly note to record **lesion extent**, visually condense all affected area to sum the extent of the lesion, then compare to the total area affected to the total visible tooth surface area, thank you.

1- Eruption status criteria

A = not visible or less than one third of the occlusal surface or of the crown length of the incisors

B= fully eruption or at least one third of the occlusal surface or of the crown length of the incisors

2- Clinical status criteria

0 = No visible enamel defect.
1 = Enamel defect, not MIH/HSPM (*hypominiralized primary second molar*).
 11 – diffuse opacites
 12 – hypoplasia
 13 – amelogenesis imperfecta
 14 – hypomineralization defect (not MIH/HSPM)

2 = White, creamy, yellow, or brown demarcated opacities.
 21 – White or creamy demarcated opacities
 22 – Yellow or brown demarcated opacities

3 = PEB. Post eruption break down.
4 = Atypical restoration.
5 = Atypical caries.
6 = Missing due to MIH/HSPM (Hypomineralized second primary molar).
7 = Cannot be scored (extensive coronal breakdown and it is impossible to determine the cause of breakdown)
(The presences of two lesions per surface, the more severe score is assigned)

3- Lesion extension/ visible surface criteria (Index teeth only, for scores 2 to 6) (Severity)

I = less than one third of the tooth surface affected.
II = at least one third but less than two thirds of the surface affected
III = at least two thirds of the tooth surface affected

(To record lesion extent, visually condense all affected area to sum the extent of the lesion, then compare to the total area affected to the total visible tooth surface area)

Sample of writing (Eruption status, Clinical status, Lesion extent)

**MAXILLA RIGHT**				**MAXILLA LEFT**			
**16**	**55**	**12**	**11**	**21**	**22**	**65**	**26**
**MANDIBLE RIGHT**				**MANDIBLE LEFT**			
**46**	**85**	**42**	**41**	**31**	**32**	**75**	**36**

Section 5: any comments:
